# A Field-Level Asset Mapping Dataset for England’s Agricultural Sector

**DOI:** 10.1038/s41597-025-05521-8

**Published:** 2025-07-15

**Authors:** Hassan Aftab Sheikh, Alok Singh, Neetu Kushwaha, Christophe Christiaen, Nataliya Tkachenko, Juan Sabuco, Ben Caldecott

**Affiliations:** 1 Smith School of Enterprise and the Environment, University of Oxford, South Parks Road, Oxford, OX1 3QY UK; 2 AI Centre of Excellence, Chief Data and AI Office, Lloyds Banking Group, London, EC2V 7HN UK

**Keywords:** Environmental social sciences, Climate-change policy

## Abstract

Agriculture sector is a major contributor to greenhouse gas emissions, yet the lack of asset-level farm data, including ownership, land use, and production, hinders effective transition finance and decarbonisation efforts. To address this gap, we developed an open-source farm-level dataset using natural language processing (NLP) and unsupervised learning, mapping farm names to spatial polygons to fill ownership and entity gaps. In England, this approach identified 117,116 farming entities with essential attributes such as addresses, land areas, crop types, production output, and geospatial coordinates. Such emerging datasets are also critical for financial instruments supporting sustainable agriculture, enabling verification of carbon credits, enhance sustainability-linked loans and improve risk assessment for climate finance.

## Background & Summary

The agricultural industry in the UK is responsible for 11% of total greenhouse gas emissions (GHG) and is a large contributor to nitrous oxide and methane emissions^1^. In 2020, agriculture accounted for 69% of the overall nitrous oxide emissions, 48% of the total methane emissions (predominantly emitted from enteric fermentation in ruminant livestock), and around 1.7% of total carbon dioxide emissions in the UK. The emissions have been mostly stagnant since 2007, despite a 16% decrease in GHG emissions from 1990-2020^2^. Despite the decrease, the UK’s agricultural industry will still face the impact of the accumulated GHG emissions. According to a report by Department for Environment, Food & Rural Affairs (DEFRA)^1^, the UK has experienced increased instances of extreme rainfall and flooding, adversely impacting crop production and supply shortages of certain domestically grown crops. The report^1^ has also noted that warmer temperatures are also likely to disrupt traditional planting and harvesting schedules as growing seasons are extended. Additionally, intensive agricultural practices have also adversely impacted biodiversity due to habitat loss and fragmentation^3^.

Climate-induced hazards and nature loss are two key risks faced by UK’s agricultural sector and have socio-economic impacts that extend beyond individual farmers to impact national food security, biodiversity, environmental quality, and water resources^4^. Policymakers are addressing these issues through frameworks such as the Common Agricultural Policy (CAP) and the Environmental Land Management (ELM) schemes. Since 2013, climate action has been one of the main objectives of the Common Agricultural Policy (CAP)^5^. The European Court of Auditors found that the European Commission had allocated €100 billion of CAP funds to support climate mitigation practices but had little impact on such emission as the CAP rarely funded schemes with high climate mitigation potential such as reducing livestock numbers, fertiliser use, and land use impacts. After Brexit, DEFRA phased out existing CAP subsidy payments and has announced an Environmental Land Management (ELM) scheme which goes beyond the production subsidy and includes land managers to provide nature positive services^6^. The ELM funding is being implemented through three schemes: (1) the Sustainable Farming Incentive (SFI) will pay farmers to maintain sustainable farming practices that protects the natural environment alongside food production, (2) Countryside Stewardship (CS) will pay for targeted actions relating to specific locations and habitats, and (3) payments for landscape recovery for longer-term large-scale projects. The SFI scheme essentially integrates sustainable farming with nature- enhancing the natural environment and reducing carbon emissions.

We understand that entities operating post-farm gate, such as food processors, distributors, and retailers, are susceptible to production shocks resulting from climate and nature shocks such as extreme weather events, biodiversity loss, and environmental pollution^4^. These disruptions can lead to increased operational costs and supply chain instability. Moreover, financial institutions with portfolios in the agricultural sector can face financial risks due to these shocks where their agricultural clients may not be able to repay loans. Many of the entities in question have also committed to net zero targets by 2050, necessitating them to disclose their emissions according to the Greenhouse Gas (GHG) Protocol^7^. This protocol differentiates emission under three heads - Scope 1 emissions: direct emissions from owned or directly controlled assets; Scope 2 emissions: indirect emissions on account of purchase of energy and Scope 3 emissions, indirect emissions on account of other purchases but not related to energy. For banks, Scope 3 emissions i.e. financed emissions comprise of the indirect impact of their investments and lending operations. Estimating these emissions is important when assessing climate-related financial risks. Financial institutions (FI)s and regulators worldwide are moving towards increased transparency in the reporting of Scope 3 emissions. FIs in Europe and North America have voluntarily adopted the emission accounting standards recommended by GHG Protocol and the Partnership for Carbon Accounting Financials (PCAF)^8^ such as Barclays^9^ and JP Morgan^10^.

Financial institutions that extend loans to farmers are accountable for financed emissions, greenhouse gas emissions generated by the agricultural activities of the borrowers they fund. This includes emissions from farming operations, such as methane and nitrous oxide from livestock and fertiliser use, as well as emissions from land-use change and deforestation. We also understand that the global demand for food, and bio-energy from agriculture is expected to rise by 60% by 2050^11^ i.e. agricultural productivity must further increase to meet this demand. This presents us with the question of how agricultural producers can maintain or increase production while reducing carbon emissions.

To understand the value and application of the proposed dataset, it is necessary to examine the specific informational demands of agricultural industry in England. First, the transition from the EU’s CAP to domestic ELM schemes requires precise field-level data to allocate payments and verify compliance. Second, transparency in land ownership is increasingly important in assessing environmental responsibility and enabling enforcement of climate-related obligations. Third, as the financial sector intensifies scrutiny of agricultural emissions under Scope 3 accounting, farm-level emissions data, linked to spatial land use, is needed for financed emissions reporting.

In England, farm holdings are around 105,200, where the majority are owner occupied (54%), followed by mixed tenure (31%), wholly tenanted (14%), and remaining 1% have undeclared tenancy^12^. Large scale corporate farmers, small-scale farmers, and financial institutions are expected to reduce the carbon footprint to be in line with the UK’s net zero goal by 2050. A dataset of farm-level assets for estimating the current state of carbon-related emissions from farming operations can help quantify emissions from production, supply chain, value chain of agricultural products, and land use change associated with expansion of agricultural activities. To address emissions throughout the value chain, and to finance sustainable agricultural practices, farm-level emissions data is useful.

The agricultural sector in England is made up of farms that vary in size, ecological context, and production practices. Although farmers are not required to report emissions, voluntary emission reporting by their stakeholders, such as clients or banks, does expect them to monitor these emissions. This can be particularly challenging for small-scale farms because of the costs involved, even when standardised carbon calculators are available. It has been discussed in previous studies^13–16^ that an ideal global open registry should incorporate key attributes such as verified ownership and tenure information, detailed land use classifications, high-resolution spatial boundaries, and productivity metrics linked to environmental and emissions data.

England’s land administration records is a collection of datasets recording land ownership, land use, and polygonal boundaries. The landscape of land administration in England is shaped by multiple institutional actors, including HM Land Registry, the Rural Payments Agency, Countryside Stewardship and DEFRA. The LR is a legal instrument that documents formal property rights, including titles, encumbrances, and transfers and the CS dataset has information of agricultural land under management within the Countryside Stewardship Agri-Environment Scheme. These agencies maintain registries of land ownership, agricultural subsidies, and land use patterns, albeit in fragmented formats. Effective environmental and financial governance requires the integration of these datasets into a unified, spatially structured system that supports emissions tracking, compliance monitoring, and subsidy management. Our work contributes toward this objective by linking fields to ownership or farm name identities and production output in a spatially explicit manner.

Previous studies that looked at modelling or mitigating farm-level emissions have used indirect estimates from existing datasets^17^, adapted IPCC estimates for specific regions^18^, or examined the connection between farm activities and management practices^19^. However, these studies have made estimates based on a small farms or model (theoretical) dataset. This is largely due to the expensive data-collection and reporting procedure. A previous study^20^ reiterated two key gaps: (1) there is no dataset of farm-level GHG emissions in Europe and (2) although farm accountancy data can serve as a proxy for calculating GHG emissions, it has limitations.

In this paper, we address the lack of an open source, spatially explicit farm-level production dataset for England for private sector stakeholders. To this end we (1) collated and matched owner data to Ordnance Survey MasterMap (OSMM)^21^ including LR^22^, and CS^23^. This was followed by (2) extracting farm information from publicly available open-source websites and Google Places API^24^; (3) geo-coding addresses to retrieve latitude, longitude information (4) performing unsupervised mapping of land parcels with farm names; (5) assigning crop to each field using Crop Map of England (CROME) map^25^; (6) estimating production numbers for each farm; and (7) validating our dataset with existing Rural Payment Agency (RPA)^26^ dataset.

The final dataset FLAME (Field level assets mapping in England) is categorised into owner or farm specific sheets. Each dataframe contains essential information on name (farm or owner), address, latitude, longitude, field area, crop type, farm type (where applicable), Standard Industrial Classification (SIC) code^27^ (where available), nature of business (where available), organic farms, (where applicable), and production. The production estimates can be used to further estimate emissions at farm level and is not calculated here since it was beyond the scope of this study. The dataset aims to provide information to stakeholders such as financial institutions and stakeholders in the agrifood value chain on modelling environmental risk and impact. For example, estimating emissions^18^, based on production numbers and emission factors; assessing flood or drought risk using geospatial hazard datasets^28–30^; or identifying opportunities for financing nature-based interventions.

## Methods

**Fig. 1:**
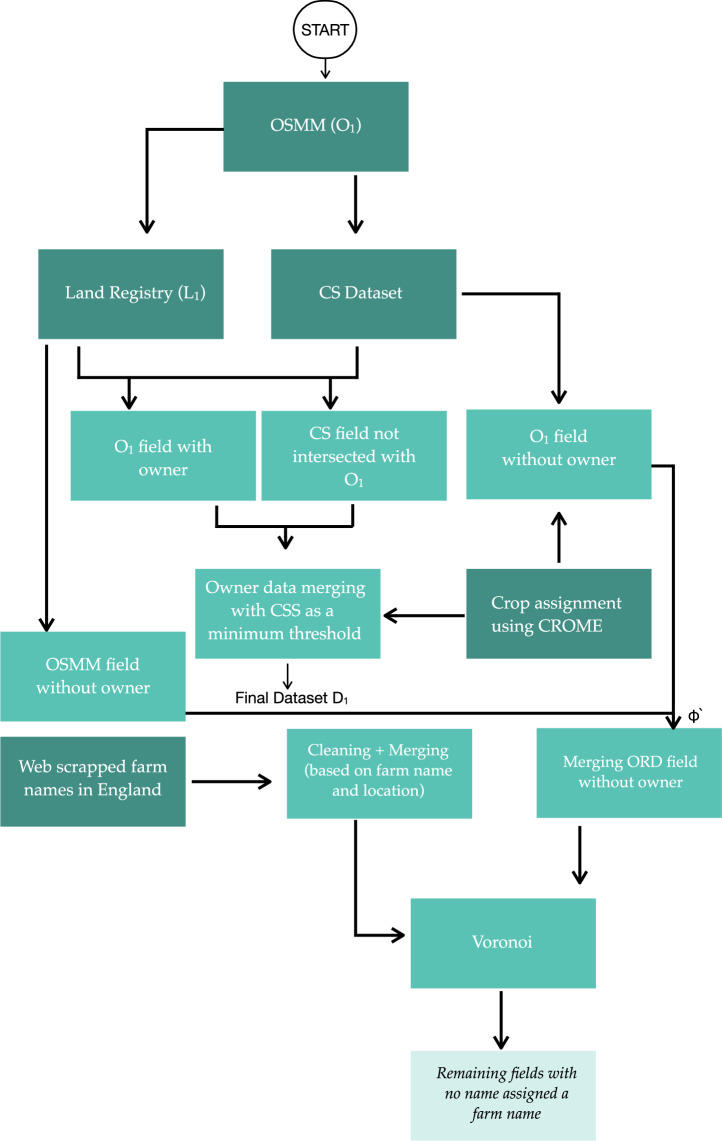
The sequential development stages of our dataset.

We employed a multi-step approach to compile the dataset for this study (Fig. 1). First, we extracted agricultural fields across England from OSMM, focusing on areas classified under the *‘agricultural land’* category (Table 1). Second, ownership and non-ownership data were gathered from the LR’s polygonal dataset, while beneficiary information was sourced from the CS dataset. We define beneficiaries as the entities or individuals that are listed as the recipients of the CS payments. Using this information, polygonal fields were mapped to their respective owners or beneficiaries. Farm names and addresses were then extracted from various sources, including DEFRA’s directory of organic producers^31^. For fields without ownership or beneficiary information, an unsupervised voronoi mapping technique was employed to associate them with the nearest known farm name. To validate our dataset, we incorporated the RPA dataset, which provides land parcel polygons used for agricultural and environmental subsidy claims. Despite limitations, such as challenges with crop classification accuracy, CROME was pivotal in assigning crop types to fields. In the following section we describe our pipeline stages in detail.

**Table 1:** Summary of Data Sources and Their Characteristics.

Dataset	Year	Coverage	Limitations
Ordnance Survey MasterMap^21^	2022	All England’s agricultural fields	May underestimate total land coverage compared to DEFRA estimates
CROME^25^	2020	Crop types in England	Does not differentiate between summer and winter crops; three years old; missing crop hexagonal data
Land Registry^22^	2023	Ownership of agricultural fields	46% of parcels lack ownership information
Companies House^27^	2023	Registered agricultural companies	Includes duplicate entries; requires filtering by SIC codes
Rural Payment Agency^26^	2023	Land parcels receiving subsidies	Used only for validation purposes; no independent data for unregistered parcels
Countryside Stewardship^23^	2023	Farmers receiving environmental incentives	Covers only 31% of agricultural land, forming a minimum threshold for farming entity assignments
Organic Farms England^31^	2022	Organic Farms in England and Wales	Lists the type of product grown at the farm

**Fig. 2:**
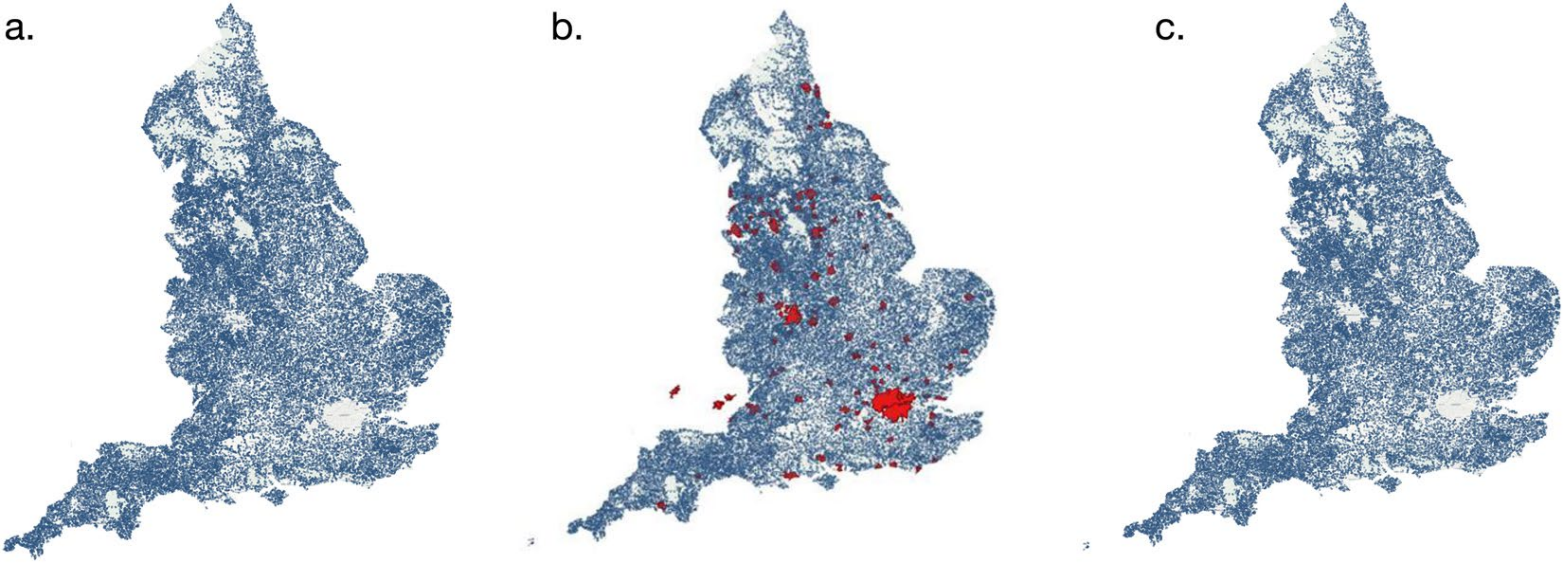
(**a**) Farms coordinates extracted from geocoding; (**b**) Major cities and towns polygons (in red) for England and (**c**) any centroids falling in built-up areas are clipped.

**Fig. 3:**
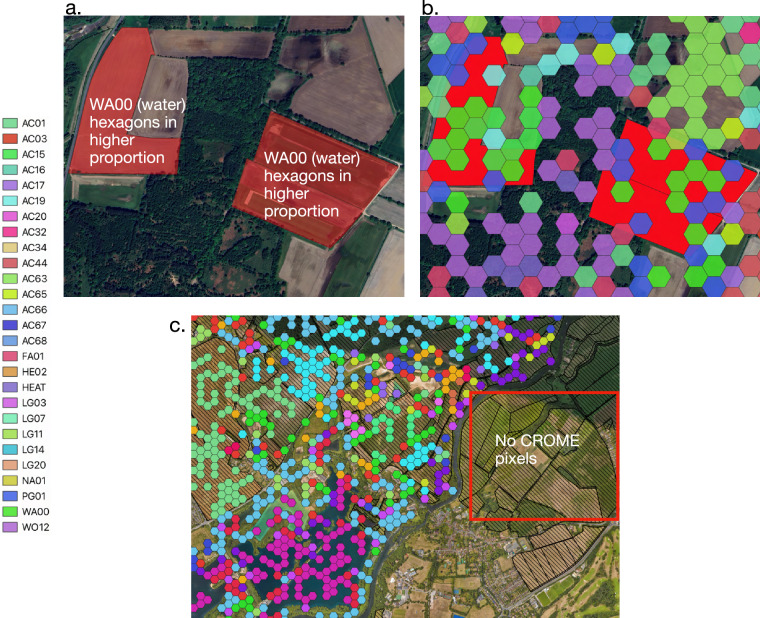
CROME hexagons illustrated on to an OSMM layer. The codes in the legend refer to different crops, more information can be found the CROME Report^25^. Location: 53.2072121,-0.7585658.

**Fig. 4:**
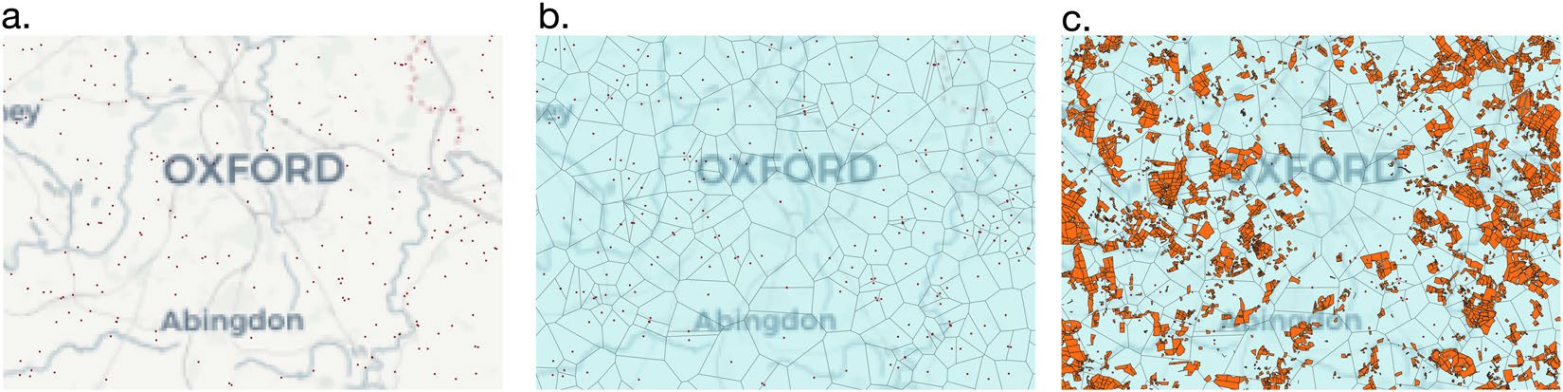
Workflow of applying Voronoi method to assign nearest lands to fields with no ownership information. (**a**) Centroids of farms extracted from web and Google Places API; (**b**) Voronoi regions generated; (**c**) Polygons with no ownership information falling into these regions are assigned land based on assumptions mentioned in section 2.

**Fig. 5:**
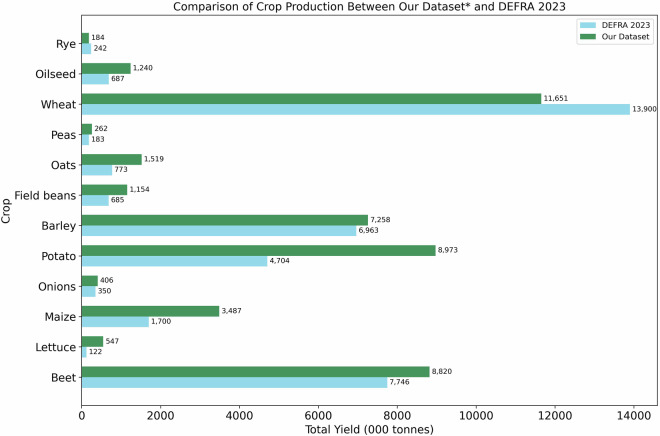
Comparison of crop production estimates between our dataset and the DEFRA (2023) statistics. *Our dataset production estimates is a combination of owner and farm-based field allocations.

### Land Registry Polygons Cleaning and Ownership Mapping

The LR data was refined by removing polygons with non-agricultural features such as buildings, urban areas, and other land types using OSMM dataset. The LR (*L*_1_) dataset was cleaned using OSMM (*O*_1_) by assigning the OSMM fields to LR polygons based on the percentage overlap between them and a three-step process was applied to further clean and categorise the dataset Table 2.

**Table 2:** Different intermediary datasets used in data merging and cleaning.

Dataset name	Description
${L}_{1}^{{\rm{with}}\,{\rm{ownership}}}$	OSMM fields linked to LR polygons with owner names (LR-OSMM)
${L}_{1}^{{\rm{without}}\,{\rm{ownership}}}$	OSMM fields linked to LR polygons without owner names
${L}_{2}^{{\rm{no}}\,{\rm{overlap}}\,{\rm{polygons}}}$	OSMM polygons that did not intersect with LR dataset
*Overlap*_*OS*2_	OSMM fields assigned to CS polygons
$O{S}_{1}^{TN}$	LR polygon assigned OSMM fields with title number information
*CS*_*A*_	OSMM fields mapped to CS polygons and remaining CS polygons that did not intersect with OSMM

Polygons with the highest percentage area overlap were stored in *Overlap*_*O*1_ as shown in Equation (1). Then, OSMM polygons that do not intersect with LR polygons were stored in ${L}_{2}^{No\,overlap\,polygons}$ as shown in Equation (1). This was followed by removing polygons with less than 10% overlap between OSMM and LR polygons and with a total area smaller than 0.05 hectares using Equation (2). The excluded polygons are stored in ${L}_{2}^{No\,overlap\,polygons}$; these polygons account for 6.5% of the total OSMM polygons. This threshold was chosen after analysis as it was observed that fields less than 0.05 ha (when overlap is <10%) were small fragments of existing OSMM polygons. In the upcoming equation, P_*_ is polygon and *A*(*) is the area of interection between polygons.

1 $$Overla{p}_{{O}_{1}}=\left\{\begin{array}{ll}{\max }_{j}\left(\frac{A({P}_{{o}_{j}}\cap {P}_{{l}_{i}})}{A({P}_{{o}_{j}})}\times 100\right), & \frac{A({P}_{{o}_{j}}\cap {P}_{{l}_{i}})}{A({P}_{{o}_{j}})}\ne 0,\\ {L}_{2}^{No\,overlap\,polygons}\leftarrow {P}_{{o}_{j}} & otherwise\end{array}\right.$$

2 $${O}_{1}^{TN}=\left\{\begin{array}{ll}{P}_{{o}_{j}}\in Overla{p}_{{O}_{1}} & Overla{p}_{{O}_{1}}({P}_{{o}_{j}})\ge 10 \% \,\text{and}\,\,Overla{p}_{{O}_{1}}({P}_{{o}_{j}}) > 0.05\,\,\text{hectares},\\ {L}_{2}^{No\,overlap\,polygons}\leftarrow {P}_{{o}_{j}}, & \,\text{otherwise}.\end{array}\right.$$

After getting polygons in ${O}_{1}^{TN}$, the ownership information was mapped using title numbers and stored in ${L}_{1}^{{\rm{with}}\,{\rm{ownership}}}$ (shown in Algorithm 1, SI). The polygons for which ownership information is not available are retained in ${L}_{1}^{{\rm{without}}\,{\rm{ownership}}}$ by merging ${L}_{2}^{No\,ownership\,polygons}$ and ${L}_{2}^{No\,overlap\,polygons}$ for farm name assignment using Voronoi.

### Mapping Countryside Stewardship (CS) Polygonal and Beneficiary Data to OSMM Fields

These farming entities, recorded under beneficiary names represent either owners or tenants. To align the CS dataset with the dimensions of the LR-OSMM dataset, the CS clusters were disaggregated into individual fields using OSMM data. This assignment is done using Equation (3) followed by Equation (4), giving us dataset *CS*_*A*_ that includes beneficiary information (owner or tenant). After this, CS polygons that did not intersect with OSMM fields were extracted and merged with *CS*_*A*_.

3 $$Overla{p}_{OS2}=\{\begin{array}{cc}{max}_{j}\left(\frac{A({P}_{{o}_{j}}\cap {P}_{{c}_{i}})}{A({P}_{{o}_{j}})},\times ,100\right),\, & \frac{A({P}_{{o}_{j}}\cap {P}_{{c}_{i}})}{A({P}_{{o}_{j}})}\ne 0,\\ Drop\,({P}_{{o}_{j}})\, & otherwise\,\end{array}$$

4 $$C{S}_{A}=\left\{\begin{array}{ll}{P}_{{o}_{j}}\in Overla{p}_{OS2} & Overla{p}_{OS2}({P}_{{o}_{j}})\ge 10 \% \,{\rm{or}}\,Overla{p}_{OS2}({P}_{{o}_{j}}) > 0.05\,\,\mathrm{hectares},\\ Drop\,({P}_{{o}_{j}}), & \,\mathrm{otherwise}.\end{array}\right.$$

### Ownership data Integration

**Fig. 6:**
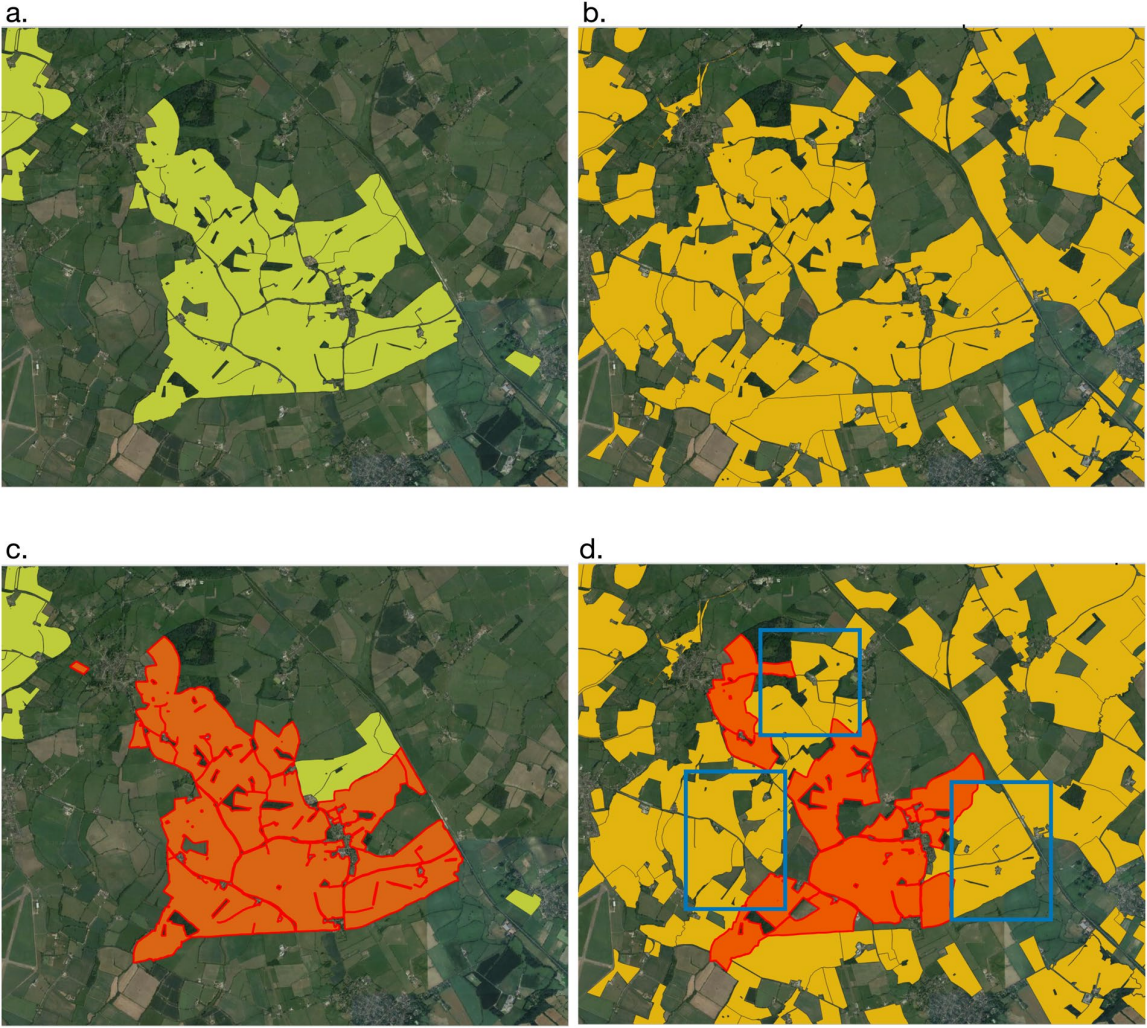
(**a**) Standard Industrial Classification- Farming polygon (**b**) Countryside stewardship polygon (**c**) Chilton Home Farms Limited (representative area in red= 961.27 ha) (**d**) Chilton under Stewardship in red: 614.825 ha.

In the previous two steps, we generated three datasets: ${L}_{1}^{{\rm{with}}\,{\rm{ownership}}}(\omega )$, ${L}_{1}^{{\rm{without}}\,{\rm{ownership}}}(\phi )$, and *CS*_*A*_(*χ*) which are checked for common *‘fid’* and updated using Equation (5). This step ensures that the polygonal area assigned to owners in *χ* is kept as a minimum threshold of an owner-associated farm entity. Figure 6c,d shows land under LR and CS respectively, where CS land can be seen as a subset of total assigned land under LR.

5 $${\omega }_{fid}^{{\prime} }={\omega }_{fid}-{\chi }_{fid}\qquad {\phi }_{fid}^{{\prime} }={\phi }_{fid}-{\chi }_{fid}$$

After cleaning dataset $\omega {\prime}$, $\phi {\prime}$ and *χ* they are assigned crop type using the approach mentioned in section *“Crop Type Assignment”*. $\omega {\prime}$ and *χ* are merged together to form dataset *D*_1_. Since, the workflow carried until now on individual fields. To aggregated the fields associated with an owner, we clustered all the fields based on owner name. Before carrying out this process, we performed cleaning over owner names to remove any spaces or lower case letters etc. After this merging, we got a Final Dataset *D*_1_. Now, to assign farm-level entity to dataset ${\phi }^{{\prime} }$, a Voronoi tessellation was applied for which farm level information is collected which is discussed below.

### Farm Data Acquisition and Preprocessing

We collected information from publicly available open-source websites and Google Places API that provide comprehensive and openly shared information relevant to farms. We employed a systemic approach by utilizing web scraping and API integration tools. The open-source websites provided freely accessible relevant farm data using web scraping techniques. For this process, we utilised Selenium^32^, a web scraping tool initially developed for automated website testing. Selenium automates browsers to load a website, retrieve its contents, and perform actions as a user would in a regular browser.

To gather information about farms from our web sources, we selected a set of keywords including: “Farms”, “Dairy Farm,” “Cattle Farm”, “Egg Supplier”, “Livestock Breeder”, “Livestock Producer,” “Organic Farms,” and “Poultry Farms”. To refine our search, we combined these keywords with geographical context. Specifically, we merged the extracted keywords with partial postcodes or city names and appended “UK” for location relevance. For example, to search for a dairy farm in the UK, we would use the keyword: “Dairy Farm in Oxford, OX4, UK”.

The extracted data includes the following details: “Place name”, “Place type”, “Address”, “Latitude”, and “Longitude”. The information from both the sources is then stored in the dataset as a separate CSV file, followed by pre-processing steps such as cleaning, deduplication, and standardization to ensure consistency and quality.

#### Data Cleaning and Geocoding

Moreover, additional cleaning was performed to standardise the dataset and prepare it for merging. This process included removing duplicate rows, extracting postcodes from address fields, eliminating unnecessary symbols, and ensuring consistent formatting across all columns. This was performed using Python’s pandas and regular expressions. By standardising the attributes across datasets from different websites ensures consistency that is useful for further data merging processes later. After extracting farm information from various sources, we identified the geolocation of each farm within the datasets. To determine their precise locations, we used the Google Geocoding API, which accepts an address as input and returns the corresponding latitude and longitude. To ensure the accuracy and robustness of the geocoding results, we randomly selected a representative sample of 5% of the total geocoded locations and manually cross-referenced the coordinates with high-resolution satellite imagery from Google Earth. We assessed the contextual alignment between the geocoded addresses and visible physical features (e.g., building clusters, roads, landmarks), confirming that the coordinates aligned with the expected address. This gave us an accuracy number of 81% on sample farm data.

#### Data Merging using Entity matching

Entity matching (EM) was a key step in the data merging process, addressing one of the main challenges of integrating datasets from various sources. The goal of EM was to identify different representations of the same real-world entity.

We utilised the Hugging Face’s Transformers library for implementation. Specifically, we utilised an SBERT^33^ NLP model based on the pre-trained *multi-qa-MiniLM-L6-cos-v1* model, which computed semantic distances between tokens using cosine similarity. The SBERT entity matching classifier uses the maximum values from cosine similarity matrices to match two entities obtained from different sources. The highest similarity scores are chosen for each record. In our analysis, we used a similarity threshold of 95% (i.e., a similarity scores above 0.95) to consider entities as matching when using SBERT.

During the merging process, we assumed that no two farms could share the same name within a given postcode. Additionally, for records lacking postcode information in their addresses, entries with duplicate farm names were removed. This step is taken because incomplete address information without a postcode increases the likelihood of inaccurate geocoding. The entire process of merging information extracted from different farm sources is illustrated in (Algorithm 2, SI).

#### Removing non-farm entities in towns and cities

The farm dataset (*D*_cleaned_) produced by the previous steps corresponded to around 65000 farms in England. However, this dataset also included non-agricultural holdings, such as shops, small businesses, or offices located in urban areas. To address this, vector boundary data for major cities and towns in England and Wales^34^ was used to filter the dataset. Farm coordinates obtained from directories that coincided with urban areas were clipped (Fig. 2). This process resulted in a cleaned dataset comprising 61,506 farms.

### Unsupervised Mapping of farm names to $\phi {\prime}$ polygons

K-means^35^ clustering is widely regarded for its performance in unsupervised mapping tasks. In our work, we initially used K-means to map fields to their nearest farms. As our problem required fixing the initial centroids (the farm locations), which we implemented using a fixed-centroid K-means approach. However, the algorithm’s inherent recalculation of centroids at each iteration proved unsuitable for our application. This recalculation caused the farm centroids to shift, resulting in misaligned field assignments. To address this issue, we incorporated a constraint to penalise deviations of the computed centroids from their fixed farm centroids. However, the penalisation process did not help us achieving our desired results. Therefore, we adopted the Voronoi^36^ algorithm, which generates regions around coordinates from dataset ${\phi }^{{\prime} }$ without assuming circular boundaries. Unlike K-means, this approach accommodates irregular geometries, making it a more effective solution for field-to-farm mapping. First, coordinate data associated with farm names (collected in Section) was used as centroids to generate Voronoi Regions as shown in Fig. 4. The generation of Voronoi region focuses on ordinary point features rather than weighted ones since as no additional parameters were available to assign weights. Therefore, each Voronoi Region was assumed to represent the fields closest to a specific farm centroid compared to any other centroid in the dataset. (See Fig. 4).

In our dataset, we collated 61,506 farm names to generate a Voronoi Region (*V*), where each centroid served as the origin of its respective region (Fig. 4b). Fields in ${\phi }^{{\prime} }$ were projected onto the *V* and farm name assignments were determined based on Equation (6). For example, if a field overlapped multiple regions by 60%, 30%, and 10%, it was assigned to the region with the 60% overlap. The entire pipeline produced two distinct datasets: *D*_1_, which represents LR-CS-OSMM mapped data containing owner information, and *D*_2_, which consists of OSMM-Voronoi data with assigned farm names.

6 $${D}_{2}={\max }_{j}\left(\frac{A\left({\phi }_{j}^{{\prime} }\cap {V}_{i}\right)}{A\left({\phi }_{j}^{{\prime} }\right)}\times 100\right)$$

### Crop Type Assignment using CROME

CROME dataset was used to assign crop types to fields defined by the OSMM field boundaries. Each field was allocated a single crop based on the greatest pixel area intersection within its boundary. Specifically, CROME pixels, each representing an area of 0.41 hectares, were analysed for their spatial distribution within a field. The crop associated with the majority of the area covered by pixels was assigned to the field. For example, in a field of 10 hectares, which had *n* pixels of *m* area of different crop classes falling, the field was assigned to the crop class with the largest cumulative pixel area overlap. The crop types were then used to assess production volumes, the methodology for which is covered in the supplementary materials.

## Data Records

The dataset is available at Zenodo^37^(reference: 15129661). The data records provide spatial information for 58,005 owner-beneficiary entities and additional coordinate information for 61,506 farm names (voronoi-mapped farms). Figure 2a shows the distribution of farm centroids in England and Fig. 2b shows the distribution of owner-assigned polygons. The attributes of the data are described in Table 3.

**Table 3:** Description of each data record in the Dataset.

Field Name	Description
Owner-beneficiary ID	Unique identifier for this dataset assigned to owners or associated -beneficiaries from Land Registry (LR) and Countryside Stewardship scheme sources.
Owner-beneficiary name	Corresponding owner of an agricultural holding according to LR or beneficiary if also under Countryside Stewardship.
Company Registration Number	Companies House registration number of the entity.
Registered Address	Address of the registered owner from LR records.
Postcode	Postcode associated with either the company address or geocoded farm address.
Second Owner	A second name appearing on the LR records.
Countryside Stewardship	Refers to the Countryside Stewardship scheme.
Owner name (LR)	Alternate name for a parcel of land in LR records compared to Stewardship parcels.
Nature of business 1-3	Agricultural-related SIC activity codes.
Nature of business type 1-3	Relevant farming activities performed by companies according to SIC registration information.
Area	Farmland area (hectares) associated with an owner-beneficiary entity or farm, derived from LR or Countryside Stewardship sources, or assigned using a Voronoi model.
Crop(s)	Crop(s) grown in the area associated with a particular owner-beneficiary entity or farm.
Total Yield	Total absolute yield (in tonnes) for a given area of farming activity, calculated using agricultural production metrics and crop area from the CROME dataset.
Grass area	Grassland area (hectares) associated with an owner-beneficiary entity or farm.
Cattle	Number of cattle assigned to an owner-beneficiary or farm (from dataset Agcensus 2016).
Sheep/Lamb	Number of sheep or lambs assigned to an owner-beneficiary or farm (from dataset Agcensus 2016).
Other Livestock	Number of other livestock assigned to an owner-beneficiary or farm (from dataset Agcensus 2016).
Farm ID	Unique identifier assigned to farm names extracted from online directory sources.
Latitude	Latitude coordinate for the farm address.
Longitude	Longitude coordinate for the farm address.
Address	Address of the farm.
Farm type	Type of farm, based on categorization from online directory sources.
Owner-beneficiary crop area	Total area of crops (in hectares) covered in the owner-beneficiary sheets of this dataset
Owner-beneficiary total yield	Total production (in tonnes/heads) covered in the owner-beneficiary sheets of this dataset
Unit	Unit in which the production yield is expressed
Farm crop area (ha)	Total area of crops (in hectares) covered in the farm sheets of this dataset
Farm crop yield	Total production (in tonnes/heads) covered in the farm sheets of this dataset
Total crop area	Total area of crops (in hectares) covered in this dataset
Total yield	Total production (in tonnes/heads) covered in this dataset

## Technical Validation

To demonstrate the quality of the final dataset, patchy and inconsistent raw data was cleaned systematically using Python libraries to minimise errors wherever possible as described in Methods section. We verified agricultural holdings allocated area information in our dataset against the publicly available RPA dataset, which encompasses 2.9 million hectares of unique fields. Validation involved mapping the Single Business Identifier (SBI), a unique nine-digit number assigned to farmers or businesses engaged in agricultural activities, against the two farm entity-level datasets, *D*_1_ and *D*_2_.

**Fig. 7:**
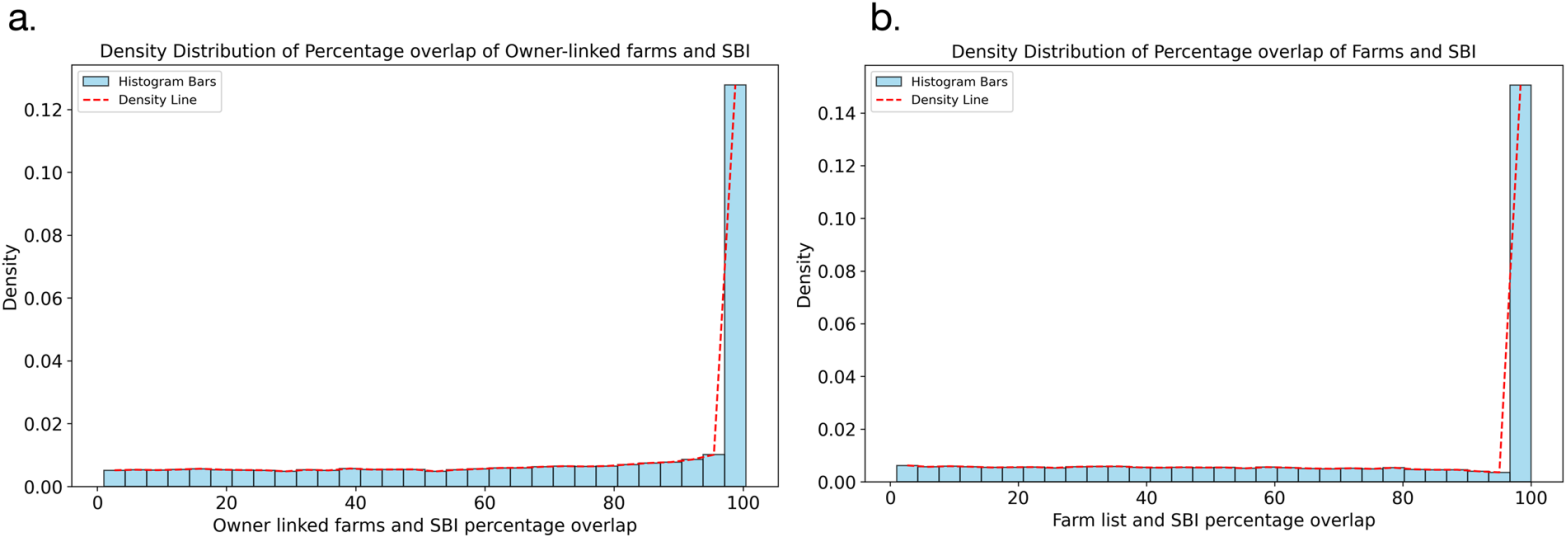
The density distribution of (**a**) Owner- and (**b**) Farm- SBI overlapped polygons. It is illustrated with a histogram (blue bars) and an overlayed density line (red dashed), where the y-axis represents the normalised density ensuring the total area under the curve equals 1.

The spatial overlap between SBI clusters and our dataset was analysed and is illustrated in Fig. 7. This analysis demonstrates that we have accurately attributed 1,073,766 hectares of owner-designated agricultural land and 560,559 hectares of farm-designated land to individual entities, aligning with the RPA’s SBI dataset. In conclusion, 17,413 out of 30,922 owner entities and 24,060 out of 42,330 farm entities—covering a combined total of 938,672 hectares achieved 80% or greater overlap within SBI parcels, drawn from a total of 2.9 million SBI records.

### Comparison of crop production between our dataset and DEFRA 2023

The robustness of our dataset was evaluated by comparing the estimated total crop production using yields from literature (see Table 2, Supplement Information) with the reported production figures from England’s DEFRA dataset (Fig. 5). Key crops such as wheat, barley, sugar beet, and field beans demonstrated concordances of 83.8% (underestimated), 96.0% (underestimated), 113.9% (overestimated), and 87.8% (underestimated), respectively, relative to national statistics. However, greater discrepancies were observed for crops such as potatoes, oilseeds, and oats, reflecting higher deviations from DEFRA estimates. These discrepancies likely arise from methodological limitations such as (1) the exclusion of crop rotations within a year and/or (2) the assignment of a single crop to a field based on highest percentage overlap despite the presence of multiple CROME pixels indicating different crops and (3) production calculation is based on literature averages, (4) the underlying OSMM geometry shapes has multiple fields aggregated under one polygon as shown in Fig. 3a, which may have meant that we assigned one crop to two different fields, and (5) CROME is better at identifying more common crops grown in England, and less accurate in identifying vegetables and fruit etc; it has also misclassified crop types such as WA00 (Water) assigned to terrestrial land in Fig. 3b.

### Limitations

There are six key limitations of our dataset. First, the Voronoi region is generated based on the assumption that the farm coordinates are at the centre of a farm. However, this may not be true since the centroid is geocoded based on open-source data. Second, for the farm-wise sheet, since we performed field-farm mapping and not field-owner, it is possible that the owner a farm may belong to an owner located farther away due to inheritance. Third, farm entities may be named differently across data sources, including varied formats, structures, and contexts. The SBERT entity matching technique is dependent on assigning a similarity score based on semantic similarity. On various occasions, we observed that we were not able to match the entities in extracted datasets due to differences in the semantics or format of the farm name. This meant that we were not successful to merge several farms where the names were not exactly the same. Fourth, the lack of information on entities’ ownership or tenancy relationship meant that we were not able to map our owner-beneficiary dataset with our farm dataset. Fifth, there is discrepancy between the timestamps of the datasets we used: CROME from 2020, LR and CSS data from 2023/2024, scraped data from 2023, and Agcensus data from 2016. Sixth, crop type based production modelling method cannot model for non-grazing livestock such as pork and poultry. This limits our ability to cover production numbers of these two in this dataset., using grasslands as a proxy for estimating livestock numbers at farm-level is a simplified version and we appreciate that there are other uses of grassland. However, producing an open-source farm-level data is a starting point for assigning GHG emissions or nature-related impacts to respective farm entities.

## Usage Notes

The data is available in an Excel Worbook file, which can be read by open source programs such as Google Sheets, Python, Microsoft Excel, Numbers etc on Zenodo^37^. Depending on the user, they can view different farm or owner-wise segregated files. Data collection and merging were implemented using Python and standard libraries like Pandas, NumPy, and geopandas. QGIS (Quantum Geographic Information System) was used for generating Voronoi regions. Additionally, we provide Python scripts for farm collection using selenium and entity matching using the NLP model. All data and Python scripts are accessible in the repository. This dataset is intended to be used to analyse a group or portfolio of individual entities in England. It should not be used to analyse individual entities in a standalone way. The dataset covers England only and draws heavily on national datasets. While similar datasets might exist in other countries, they might not be exactly identical in scope. To replicate the methodology for other countries, adjustments might have to be made, taking into account availability and data features of national datasets.

## Supplementary information

Supplementary Information

## Data Availability

The code for this paper is open-source and available at Github.
