# Supplementary material for: A Field-Level Asset Mapping Dataset for England’s Agricultural Sector
Source: Sci Data. 2025 Jul 15;12:1240. doi: 10.1038/s41597-025-05521-8 (PMC12263996; doi:10.1038/s41597-025-05521-8)
Supplement: Supplementary file 1 — Supplementary Information [file 41597_2025_5521_MOESM1_ESM.pdf]

FLAME Dataset: Farm-Level Assets Mapping in England

1 Introduction

This supplementary document provides additional details related to the methodology employed in this study and the data produced in this study that complement our database presented in the main manuscript.

2 Production estimates

The quantification of total production per field was calculated using yield values from the John Nix Pocketbook[1] and FAO UK average[2] production statistics 2. These yield figures, representing the output per unit area, formed the basis of the estimation methodology. For each crop, production was calculated by combining these yield values for each crop from CROME (see Table 1) with the specific field area associated with each farm in the non-owner and owner-company datasets. By multiplying the production per hectare by the corresponding field area, an estimate of crop production was derived for each field. This method was applied consistently across all farm and company-owned land parcels, which are likely to represent distinct farming entities. The formula for this calculation is as follows:

$$Production_{crop_i} = Yield_{crop_i} * Area_{crop_i} \quad (1)$$

2.1 Livestock number calculation

In this analysis, 'Grass' from crop type is used as a proxy for estimating livestock populations, specifically sheep, cattle, and horses as these animals are predominantly reared on grassland. To estimate, we used data from the 2016 Agricultural Census of the UK (AgCensus) for England[3].

2.1.1 Cattle, Sheep, and Horses

In England, the AgCensus provides estimates of agricultural activities and related data at a 5 km x 5 km resolution. To attribute livestock at the field level, we integrated the AgCensus and OSMM datasets. Grasslands were assumed to serve as proxies for shared grazing areas for livestock, including cattle, sheep, lambs, and horses. To estimate livestock distribution, all grass fields within a 5 km x 5 km grid were aggregated for each farm-level entity. The total livestock population within each grid was then proportionally redistributed among the grass fields based on their respective areas as shown in Figure 1.

Table 1. Crops description as per CROME dataset (2020)[4]

Land Cover Description	LUCODE	Land Use Description
Cereal Crops	AC01	Spring Barley
	AC03	Beet
	AC04	Borage
	AC05	Buckwheat
	AC06	Canary Seed
	AC07	Carrot
	AC09	Chicory
	AC10	Daffodil
	AC14	Hemp
	AC15	Lettuce
	AC16	Spring Linseed
	AC17	Maize
	AC18	Millet
	AC19	Spring Oats
	AC20	Onions

Land Cover Description	LUCODE	Land Use Description
	AC22	Parsley
	AC23	Parsnips
	AC24	Spring Rye
	AC26	Spinach
	AC27	Strawberry
	AC30	Spring Triticale
	AC32	Spring Wheat
	AC34	Spring Cabbage
	AC35	Turnip
	AC36	Spring Oilseed
	AC37	Brown Mustard
	AC38	Mustard
	AC41	Radish
	AC44	Potato
	AC45	Tomato
	AC50	Squash
	AC52	Siam Pumpkin
	AC58	Mixed Crop-Group 1
	AC59	Mixed Crop-Group 2
	AC60	Mixed Crop-Group 3
	AC61	Mixed Crop-Group 4
	AC62	Mixed Crop-Group 5
	AC63	Winter Barley
	AC64	Winter Linseed
	AC65	Winter Oats
	AC66	Winter Wheat
	AC67	Winter Oilseed
	AC68	Winter Rye
	AC69	Winter Triticale
	AC70	Winter Cabbage
	AC71	Coriander
	AC72	Corn Gromwell
	AC74	Phacelia
	AC81	Poppy
	AC88	Sunflower
	AC90	Gladioli
	AC92	Sorghum
	AC94	Sweet William
	AC100	Italian Ryegrass
	CA02	Cover Crop
Leguminous Crops	LG01	Chickpea
	LG02	Fenugreek
	LG03	Spring Field Beans
	LG04	Green Beans

Land Cover Description	LUCODE	Land Use Description
	LG06	Lupins
	LG07	Spring Peas
	LG09	Cowpea
	LG08	Soya
	LG11	Lucerne
	LG13	Sainfoin
	LG14	Clover
	LG15	Mixed Crops–Group 1 Leguminous
	LG16	Mixed Crops–Group 2 Leguminous
	LG20	Winter Field Beans
	LG21	Winter Peas
Energy Crop	SR01	Short Rotation Coppice
Grassland	FA01	Fallow Land
	HE02	Heathland and Bracken
	HEAT	Heather
	PG01	Grass
Non-Agricultural Land	NA01	Non-vegetated or Sparsely-Vegetated Land
Water	WA00	Water
Trees	TC01	Perennial Crops and Isolated Trees
	NU01	Nursery Crops
	WO12	Trees and Scrubs, Short Woody Plants, Hedgerows
Unknown Vegetation or Mixed Vegetation	AC00	Unknown or Mixed Vegetation

Table 2. Agricultural production metrics.

Crop/Item	Yield	Unit	Category	Source
Spring Barley	5.75	Tonnes per hectare	Crops	John Nix Pocketbook
Beet	77	Tonnes per hectare	Crops	John Nix Pocketbook
Borage	0.4	Tonnes per hectare	Crops	John Nix Pocketbook
Carrot	62.3974	Tonnes per hectare	Crops	FAO
Chicory	23.3448	Tonnes per hectare	Crops	FAO
Hemp	7.5	Tonnes per hectare	Crops	John Nix Pocketbook
Lettuce	23.3448	Tonnes per hectare	Crops	FAO
Spring Linseed	1.75	Tonnes per hectare	Crops	John Nix Pocketbook
Maize	7.5	Tonnes per hectare	Crops	John Nix Pocketbook
Millet	3	Tonnes per hectare	Crops	John Nix Pocketbook
Spring Oats	5.6	Tonnes per hectare	Crops	John Nix Pocketbook
Onions	40.5989	Tonnes per hectare	Crops	FAO
Spring Rye	5.5	Tonnes per hectare	Crops	John Nix Pocketbook
Strawberry	20	Tonnes per hectare	Crops	John Nix Pocketbook
Spring Triticale	4.5	Tonnes per hectare	Crops	John Nix Pocketbook

Crop/Item	Yield	Unit	Category	Source
Spring Wheat	6.2	Tonnes per hectare	Crops	John Nix Pocketbook
Spring Cabbage	24.967	Tonnes per hectare	Crops	FAO
Turnip	62.3974	Tonnes per hectare	Crops	FAO
Spring Oilseed	2.28	Tonnes per hectare	Crops	John Nix Pocketbook
Brown Mustard	1	Tonnes per hectare	Crops	John Nix Pocketbook
Potato	50.4	Tonnes per hectare	Crops	John Nix Pocketbook
Tomato	350.3544	Tonnes per hectare	Crops	FAO
Winter Barley	6.7	Tonnes per hectare	Crops	John Nix Pocketbook
Winter Linseed	2	Tonnes per hectare	Crops	John Nix Pocketbook
Winter Oats	6.1	Tonnes per hectare	Crops	John Nix Pocketbook
Winter Wheat	8.6	Tonnes per hectare	Crops	John Nix Pocketbook
Winter Oilseed	3.5	Tonnes per hectare	Crops	John Nix Pocketbook
Winter Rye	5.5	Tonnes per hectare	Crops	John Nix Pocketbook
Winter Triticale	4.5	Tonnes per hectare	Crops	John Nix Pocketbook
Winter Cabbage	24.967	Tonnes per hectare	Crops	FAO
Sunflower	2.5	Tonnes per hectare	Crops	John Nix Pocketbook
Spring Field Beans	3.9	Tonnes per hectare	Crops	John Nix Pocketbook
Green Beans	5.2834	Tonnes per hectare	Crops	FAO
Lupins	3	Tonnes per hectare	Crops	John Nix Pocketbook
Spring Peas	4	Tonnes per hectare	Crops	John Nix Pocketbook
Soya	2.2	Tonnes per hectare	Crops	John Nix Pocketbook
Winter Field Beans	4.3	Tonnes per hectare	Crops	John Nix Pocketbook
Winter Peas	4	Tonnes per hectare	Crops	John Nix Pocketbook

Algorithm 1: Cleaning Land Registry Data ($L_1(P_l)$) using OSMM Data ($O_1(P_o)$) and Owner Title Number (Γ)

Input: P_l, P_o, Γ
Output: $L_1^{\text{with ownership}}$ and $L_1^{\text{without ownership}}$
Step 1: Cleaning P_l using P_o;
$Overlap_{O_1} \leftarrow$ Intersection(P_{o_j}, P_{l_i});
$L_2^{\text{No overlap polygons}} \leftarrow$ polygons in P_{o_j} not intersecting with P_{l_i};
$Overlap_{O_1}$["Overlap%"] $\leftarrow$ compute_percent_overlap(P_{o_j}, P_{l_i});
$Overlap_{O_1}$["Overlap_area"] $\leftarrow$ compute_area_overlap(P_{o_j}, P_{l_i});
$Condition \leftarrow (Overlap_{O_1}$["Overlap%"] $\geq 10)$ & $(Overlap_{O_1}$["Overlap_area"] $> 0.05)$;
$O_1^{TN} \leftarrow Overlap_{O_1}[Condition]$;
$L_2^{\text{No overlap polygons}} \leftarrow Overlap_{O_1}[\sim Condition]$;
Step 2: Assign owner to O_1^{TN} using Title Number Γ;
if O_1^{TN}["Title Number"] $\in \Gamma$ **then**
  $L_1^{\text{with ownership}} \leftarrow$ concat$(O_1^{TN}$, owner_info$)$;
else
  $L_2^{\text{No ownership polygons}} \leftarrow O_1^{TN}$;
  $L_1^{\text{without ownership}} \leftarrow$ concat$(L_2^{\text{No ownership polygons}}, L_2^{\text{No overlap polygons}})$;
Result: $L_1^{\text{with ownership}}$ and $L_1^{\text{without ownership}}$

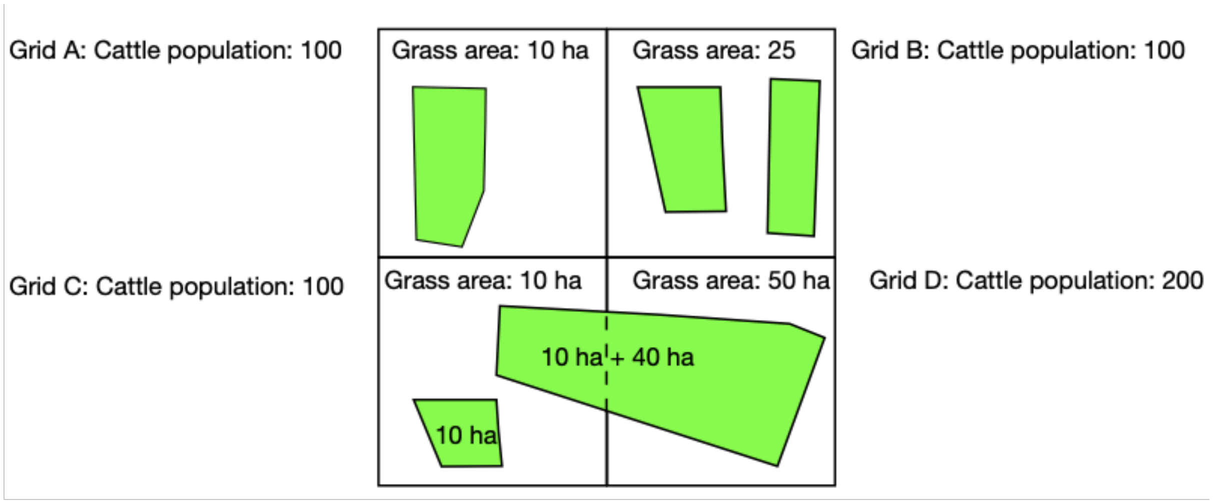

Figure 1. Illustrates the formation of our attribution algorithm through agCensus dataset[3] as a proxy. The size of a field, measured in hectares, has been used as a proxy for livestock. We identify grass fields within a given 5 * 5 km grid and calculate densities. In case where a field is intersecting in two or grids, we assign the total field area to the grid with higher intersection such as to Grid D in this case. (A) The density of cattle per hectare of grass field is 10 cattle/ha; (B): 4 cattle/ha; (C) 10 cattle/ha (D) 4 cattle/ha.

Algorithm 2: Dataset Cleaning and Merging with n Datasets (Iterative Process)

Input: Datasets $D_1, D_2, \ldots, D_n$ with columns [FarmName, Latitude, Longitude, Address, Postcode]
Output: Farm Dataset D_{cleaned}

Step 1: Remove Duplicates Based on FarmName, Latitude, and Longitude;
foreach $D_i \in \{D_1, D_2, \ldots, D_n\}$ **do**
  $D_i \leftarrow \text{RemoveDuplicateRows}(D_i, \text{columns} = [\text{FarmName}, \text{Latitude}, \text{Longitude}])$;
Step 2: Divide Datasets into Two Parts;
$D_{\text{postcode}} \leftarrow \text{FilterByPostcode}(D_1, D_2, \ldots, D_n)$;
$D_{\text{no\_postcode}} \leftarrow \text{FilterWithoutPostcode}(D_1, D_2, \ldots, D_n)$;
Step 3: Initialize Merged Datasets;
$D_{\text{merged\_postcode}} \leftarrow \emptyset$;
$D_{\text{merged\_no\_postcode}} \leftarrow \emptyset$;
Step 4: Handle Datasets with Postcodes;
foreach $D_i \in D_{postcode}$ **do**
  $D_{\text{merged\_postcode}} \leftarrow D_{\text{merged\_postcode}} \cup D_i$;
  $D_{\text{merged\_postcode}} \leftarrow \text{GroupBy}(D_{\text{merged\_postcode}}, \text{'Postcode'})$;
  $D_i \leftarrow \text{GroupBy}(D_i, \text{'Postcode'})$;
  foreach $P \in Postcodes$ **do**
    foreach $r_1 \in D_{merged\_postcode}[P]$ **do**
      foreach $r_2 \in D_i[P]$ **do**
        if $SBERTSimilarity(r_1[FarmName], r_2[FarmName]) > 95\%$ **then**
          remove r_1 from $D_{\text{merged\_postcode}}$; keep r_2 from D_i;
  $D_{\text{merged\_postcode}} \leftarrow \text{Merge}(D_{\text{merged\_postcode}}, D_i)$;
Step 5: Handle Datasets without Postcodes;
foreach $D_i \in D_{no\_postcode}$ **do**
  foreach $r_1 \in D_{merged\_no\_postcode}$ **do**
    foreach $r_2 \in D_i$ **do**
      if $SBERTSimilarity(r_1[FarmName], r_2[FarmName]) > 95\%$ **then**
        remove r_1 from $D_{\text{merged\_no\_postcode}}$; keep r_2 from D_i;
  $D_{\text{merged\_no\_postcode}} \leftarrow \text{Merge}(D_{\text{merged\_no\_postcode}}, D_i)$;
Step 6: Final Merge;
$D_{\text{cleaned}} \leftarrow \text{Merge}(D_{\text{merged\_postcode}}, D_{\text{merged\_no\_postcode}})$;
Result: Farm dataset D_{cleaned}

References

1. Nix, J., Hill, G., Johnson, P. & Lampkin, N. *John Nix Pocketbook for Farm Management* (Melton Mowbray: Agro Business Consultants Ltd., 2022), 51st edn.
2. Food and Agriculture Organization of the United Nations. Fao statistics. http://www.fao.org/statistics/en (2022).
3. Agricultural Census. Agricultural census data for england, 2016 (2016).
4. DEFRA. Crop map of england dataset. https://environment.data.gov.uk/dataset/cc389fe9-f026-4b20-a80f-f424ee833ea6 (2020).
